# Quantitative Trait Locus Mapping and Identification of Candidate Genes for Resistance to Fusarium Wilt Race 7 Using a Resequencing-Based High Density Genetic Bin Map in a Recombinant Inbred Line Population of *Gossypium barbadense*

**DOI:** 10.3389/fpls.2022.815643

**Published:** 2022-03-10

**Authors:** Wanli Han, Jieyin Zhao, Xiaojuan Deng, Aixing Gu, Duolu Li, Yuxiang Wang, Xiaoshuang Lu, Qianli Zu, Qin Chen, Quanjia Chen, Jinfa Zhang, Yanying Qu

**Affiliations:** ^1^Engineering Research Centre of Cotton, Ministry of Education/College of Agriculture, Xinjiang Agricultural University, Ürümqi, China; ^2^Department of Plant and Environmental Sciences, New Mexico State University, Las Cruces, NM, United States

**Keywords:** *Gossypium barbadense*, recombinant inbred lines, genome resequencing, single-nucleotide polymorphism (SNP), quantitative trait locus (QTL), Fusarium wilt (FOV7), candidate genes

## Abstract

Fusarium wilt caused by *Fusarium oxysporum* f. sp. *vasinfectum* (FOV) is one of the most destructive diseases in cotton (*Gossypium* spp.) production, and use of resistant cultivars is the most cost-effective method managing the disease. To understand the genetic basis of cotton resistance to FOV race 7 (FOV7), this study evaluated a recombinant inbred line (RIL) population of 110 lines of *G. barbadense* from a cross between susceptible Xinhai 14 and resistant 06-146 in eight tests and constructed a high-density genetic linkage map with resequencing-based 933,845 single-nucleotide polymorphism (SNP) markers covering a total genetic distance of 2483.17 cM. Nine quantitative trait loci (QTLs) for FOV7 resistance were identified, including *qFOV7-D03-1* on chromosome D03 in two tests. Through a comparative analysis of gene expression and DNA sequence for predicted genes within the QTL region between the two parents and selected lines inoculated with FOV7, *GB_D03G0217* encoding for a calmodulin (CaM)-like (CML) protein was identified as a candidate gene. A further analysis confirmed that the expression of *GB_D03G0217* was suppressed, leading to increased disease severity in plants of the resistant parent with virus induced gene silencing (VIGS).

## Introduction

Cotton (*Gossypium* spp.) is the most important fiber crop for the textile industry in the world. In addition, it is also an important source of feed, food and biofuel components (Fang and Percy, 2015). *G. hirsutum* (Upland cotton) is a tetraploid cotton species and is grown in more than 80 countries, producing 97% of world cotton due to its high yield and wide adaptation. Its closed related another tetraploid *G. barbadense* (extra-long staple, Egyptian, Pima or Sea-Island cotton) is grown for high quality fibers with long, strong and fine parameters (Zhang et al., 2014). However, due to its low yield potentials and requirements for warm and dry weathers, *G. barbadense* is only grown in limited production areas in about a dozen of countries including China, India, Uzbekistan, Tajikistan, Turkmenistan, Spain, Egypt, Israel, Sudan, Peru, and the United States^1^. China ranks the third after the United States and India in production of the extra-long staple *G. barbadense*, which is grown in the Xinjiang Autonomous Region, a Northwest province.

There are various limiting factors that threaten cotton production (Hillock, 1992). Fusarium wilt (FW), caused by the fungus *Fusarium oxysporum* f. sp. *vasinfectum* (FOV), is one of the most serious diseases of cotton (Zhang et al., 2015; Sanogo and Zhang, 2016; Bell et al., 2019). FOV is a soil-borne plant fungal pathogen that causes Fusarium wilt through root infection in many plant species, including important economic crops such as cotton, cabbage, banana, watermelon and tomato (De Cal et al., 2000; Galindo and Deyholos, 2016; Xing et al., 2016; Dale et al., 2017; Li et al., 2017; Rana et al., 2017; Upasani et al., 2017). This pathogen exists in the soil and crop residues in the form of mycelia, chlamydia or small sclerotia and can survive in the soil for 5–10 years before causing infection (Galindo and Deyholos, 2016). FW usually develops after the seedling stage, and its visual symptoms include leaf chlorosis and necrosis, wilting, vascular tissue discoloration, and ultimately plant death (Ma and Zhang, 1986). In cotton, 8 nominal races have been reported based on pathogenicity tests using a set of differential plant hosts, including races 1 and 2 in the United States and Tanzania, race 3 in Egypt, Sudan and Israel, race 4 in India and the United States (Kim et al., 2005; Halpern et al., 2019; Zhu et al., 2020), race 5 in Sudan, race 6 in Brazil and Paraguay, and races 7 and 8 in China (Davis et al., 2006). However, races 3 and 5, as well as races 4 and 7 were later found to be indistinguishable between one another based on DNA, genetic and morphological analyses (Davis et al., 2006; Bell et al., 2019; Halpern et al., 2020; Zhu et al., 2021). Although both races 7 and 8 were reported in China, race 7 is more widespread and more virulent than race 8 which is limited in a very narrow area. Among different strategies managing FOV in cotton production, breeding and employment of resistant cultivars has been the only cost-effective method (Zhang et al., 2015; Sanogo and Zhang, 2016).

Most *G. barbadense* cultivars and accessions in Chinese cotton germplasm collection are susceptible or highly susceptible to FOV7 (Zhang et al., 2015). Zhu et al. (2010) mapped a single dominant resistance gene on chromosome D11 (c21) in early segregating populations from a *G. barbadense* cross of susceptible Xinhai 21 × highly resistant HK 237 based on simple sequence repeat (SSR) markers. However, no follow-up validation was reported. In the United States, a major dominant resistant gene (*FOV1*) to race 1 on chromosome D07 (c16) was identified and mapped in Pima S-7 (*G. barbadense*) using SSR markers (Wang and Roberts, 2006; Ulloa et al., 2011). Another major resistance gene (*FOV4*) conferring resistance to race 4 was identified in resistant Pima S-6 and mapped to chromosome D02 (c14), based on also SSR markers (Ulloa et al., 2013).

In China, genetic studies and breeding for FOV7 resistance has mainly been focused on Upland cotton since the 1950s, when two highly resistant lines- Chuan 52–128 and Chuan 57–681 were developed through repeated selections in FOV7-infested fields from highly susceptible Delfos 531 and Deltapine 15, respectively (Zhang et al., 2015). These two resistant Upland cotton lines have become the donors for numerous resistant commercial cultivars developed in the 1960s and onward. Through quantitative and qualitative (Mendelian) genetic analyses (Feng et al., 1998), the two sources of resistance were found to carry two different resistance genes (*F*_*W*1_ and *F*_*W*2_). In a subsequent SSR-based marker analysis, a dominant resistance gene (*F*_*W*_^R^), presumably one of the two major resistance genes (*F*_*W*1_ and *F*_*W*2_), was mapped to chromosome D03 (c17), using two F_2:3_ populations (Wang et al., 2009). Most recently, through a genome-wide association study (GWAS) on 290 Chinese Upland accessions followed with a CRISPR/Cas9-mediated knockout, *Gh_D03G0209* (named *GhGLR4.8*) on D03 encoding a GLUTAMATE RECEPTOR-LIKE (GLR) protein was identified as the candidate gene for the resistance gene *Fov7* (Liu et al., 2021). *Fov7* is presumably one of the two major resistance genes identified and named earlier by Feng et al. (1998). However, whether there exist the same resistance genes conferring resistance to FOV7 in *G. barbadense* is currently unknown.

Because cotton responses to FOV infections can be quantitatively measured through a rating system based on visual disease severity (Sanogo and Zhang, 2016), genetic studies on FOV resistance in cotton have been often performed using quantitative genetic approaches such as diallel analysis and generation mean analysis (for a review, see Zhang et al., 2015). With the advent of molecular markers, quantitative trait locus (QTL) mapping has been used to locate QTLs for FOV resistance on chromosomes, resulting in reporting of numerous QTLs associated with FOV resistance (Said et al., 2013, 2015a,2015b; Zhang et al., 2015; Abdelraheem et al., 2017). However, QTL mapping for FOV resistance using linkage analysis and GWAS has been almost exclusively focused on Upland cotton. There have been a few reports using SSRs in genomic studies of *G. barbadense* (Abdelraheem et al., 2013, 2015, 2020; Wang et al., 2013), and no high density linkage map has been published for *G. barbadense* because a low level of polymorphisms in SSR markers within the species.

The genomes of 3–79, Hai 7124 and Xinhai 21 of *G. barbadense* have been recently sequenced with updates in China (Hu et al., 2019; Wang et al., 2019), providing important genomic resources for studying the genetics and genomics of this important cultivated cotton species in order to accelerate breeding using marker-assisted selection (MAS) or genomic selection (GS). However, the major QTLs for FOV7 in *G. barbadense* are currently not well understood. To identify QTLs and discover candidate genes related to FOV7 resistance in *G. barbadense*, our study took advantage of the genome sequence information of *G. barbadense* to construct the first high-quality, high-density genetic linkage map in *G. barbadense* based on genome resequencing of 110 recombinant inbred lines (RILs) from a cross of FOV7-susceptible Xinhai 14 and FOV7-resistant 06-146. The map was used to identify FOV7 resistance QTLs. A stable QTL on chromosome D03 was identified, and genes within the QTL region were further evaluated through RNA-seq of the two parents and quantitative RT-PCR (qRT-PCR) analysis of selected RILs. A candidate gene was validated through virus-induced gene silencing (VIGS). This study combined genome resequencing and RNA-seq in a RIL population, providing new knowledge for understanding the genetic and genomic basis of resistance to FOV7 in *G. barbadense*.

## Materials and Methods

### Plant Materials

A single-seeded descent method was used to advance F_2_ plants from a cross between the FOV7-susceptible *G. barbadense* cultivar Xinhai 14 and the FOV7-resistant 06-146. As a result, 110 F_2:7_ RILs were produced and used in this study.

### Evaluation for FOV7 Resistance and Data Analysis

The two parents and 110 RIL lines were grown in eight tests including seven field tests and one laboratory test between 2011 and 2018. In 2011–2012, four field tests were first conducted including two field tests each year at Experimental Base, Xinjiang Academy of Agricultural Sciences, Alar, Xinjiang, and Experimental Farm, Xinjiang Korla Academy of Agricultural Sciences, Alar, Xinjiang. This was followed by three field tests in 2016–2018 each year at the same site of Alar. Single-row plots were used with the plot length of 3 m, the row spacing of 0.35 m, and the plant spacing of 0.10 m. For each of the above seven field tests, a randomized complete block design (RCBD) with three replications was used to arrange all the experimental units (i.e., the two parents and 110 RILs). Seeds were hand planted in mid-April each year, and crop management practices followed local recommendations.

In 2017, a laboratory test using artificial inoculation was performed using the same RCBD with three replications on campus of Xinjiang Agricultural University, Urumqi, Xinjiang. Seeds were sown to pots filled with a mixture of vermiculite and sterilized farm soil (in a ratio of 1:2) in a manner of 10 holes per pot with two seed per hole on May 3rd,2017. The daily temperature in the laboratory ranged between 25.0 and 28.0°C, with supplementary lights provided. At the cotyledon stage, plants were artificially inoculated with 50 ml of FOV7 inoculum (with the concentration of 1 × 10^6^ conidia ml^–1^) via the root-wounding method (Pei et al., 2019). Non-inoculated pots for the RILs were used as control. FOV7-caused disease symptoms were first observed at the 2-leaf stage.

In all the evaluations for FOV7 resistance, a rating scale of 0 to 4 based on foliar disease severity was used to evaluate each plant, as the following: 0 = healthy, with no disease symptoms; 1 = 25.0% of the leaf area exhibited disease symptoms; 2 = 25.1–50.0% of the leaf area exhibited disease symptoms or plants were slightly dwarfed in stature; 3 = 50.1–75.0% of the leaf area exhibited disease symptoms or plants severely dwarfed in stature; and 4 = > 75.0% of the leaf area exhibited disease symptoms or plants completely defoliated or died (Sanogo and Zhang, 2016). For the seven field tests, evaluation for FOV7 resistance was conducted during flowering or boll development stage (i.e., mid-July to early October) each year. For the laboratory test, screening was completed on May 23th, 2018, i.e., 20 days after inoculation FOV7 (Supplementary Table 1).

The average disease severity rate (DSR), i.e., the sum of DSRs divided by the number of plants, was calculated among the replicates of each line. The describe function in the Hmisc package of R was used to perform a descriptive statistical analysis of the RIL population. Analysis of variance and generalized heritability determination in the parents and RILs were performed in R. If the *F* test was significant, then a multiple comparison least significant difference (LSD) test (with *P* < 0.05 indicating significance) was used (Li et al., 2016).

### DNA Library Preparation and Resequencing

In July 2017, young leaves of the two parents and 110 RILs were collected and stored at −70°C. Using a miniprep method (Zhang and Stewart, 2000), genomic DNA for each line was extracted and cleaned. The sequencing library was constructed based on the follow steps: the quantified DNA samples were randomly sheared by ultrasonic disruption, and DNA fragments were repaired with an addition of A at the 3′ end. Followed by addition of sequencing adapters, the ligated DNA fragments were purified and amplified by PCR using primers designed from the adaptors. Finally, the library for each line was sequenced using the Illumina sequencing platform.

### Single-Nucleotide Polymorphism (SNP) Identification of Sequencing Data

The raw sequence reads obtained by sequencing were filtered to obtain clean reads, by removal of adapter sequences and low quality reads with N bases greater than 10%. The clean paired-end resequencing reads were mapped to the reference genome of *G. barbadense* Hai 7124 (Hu et al., 2019) using the Burrows–Wheeler Alignment (BWA) software (Li and Durbin, 2009). After duplicate reads were removed by the Picard’s Mark Duplicate tool, the GATK software was used to identify SNPs and Indels (Simona et al., 2017).

### Construction of a High Density Genetic Map

The linkage map of the RIL population was constructed using HighMap software (Liu et al., 2014). SMOOTH was used to correct errors based on parental genotype contributions, and the k-nearest neighbor algorithm was used to correct incorrect genotypes (Hans et al., 2005). Map distance was calculated using the Kosambi mapping function. The chi-square test was used to identify SNPs deviated from the expected 1:1 ratio for SNPs in the RIL population. Polymorphic SNPs with significant segregation distortion (*P* < 0.01) were removed from linkage analysis.

The SNP markers in the linkage map were further aligned to the reference genome using the local BLAST method. The parental markers with a sequencing depth of 10 × or higher were used to select SNPs in the RIL population, and a marker that was not mapped on a chromosome in the parents was removed. Among the resulted 933,845 SNPs, a moving window of 15 SNPs with 11 or more SNPs of parental type aa or bb was considered homozygous aa or bb type. Otherwise, it was deemed an ab type. When there was a SNP typing change along a chromosome in any line, a recombination breakpoint occurred. SNPs between recombination breakpoints were classified into recombination bins. A recombination bin was defined as a chromosomal segment with no genetic recombination. Finally, a representative SNP within each bin was used to construct a genetic map. Bins within a less than 10 kb region were combined.

### Quantitative Trait Locus Mapping

The mean phenotypic DSR values for RILs in 8 tests were used for QTL mapping with the R-3.6.2 software package “qtl.” A composite interval mapping method was employed to locate QTLs for FOV7 resistance (Karl and Saunak, 2009). A stringent LOD threshold was calculated through a permutation test. The parameters for QTL mapping procedure were set as follows: calculation time of 1,000 times, Type I error *P* value at 0.05, PIN (probability level of entering the model) at 0.001, and mapping step length of 1.0 cM. If QTLs identified in two or more tests with confidence intervals (CI, 95%) overlapped, they were considered the same and stable QTL. The CI for each QTL was set as the location distance interval corresponding to a one LOD drop from both sides of the peak.

### Identification of Candidate Genes Through RNA-Seq and Quantitative RT-PCR

To identify potential candidate genes, BLAST was used to extract gene sequences within stable QTL regions based on the reference genome (Hu et al., 2019), and predicted genes in the associated regions were compared with these in different genome databases- the GO and KEGG (Du et al., 2020). To analyze the general expression pattern of the candidate genes, the transcriptome (i.e., RNA-seq) from the two parents (Xinhai14 and 06-146) and selected highly resistant (HR) and highly susceptible (HS) RILs were performed on a cDNA library constructed using 10 μg of total RNA extracted from each biological sample of each genotype (Yao et al., 2019). Briefly, seeds for these lines were grown in pots and inoculated with FOV7 conidia, as described earlier. The roots, stems and young leaf tissues for each genotype were collected at 0, 4, 8, 12, 24, and 48 h post-inoculation. Three biological samples for each line were separately harvested for RNA extraction. A total RNA extraction kit (Tiangen, China) was used to extract RNA from the biological samples. Poly-adenylated RNA was purified and concentrated with oligo (dT)-conjugated magnetic beads (Invitrogen, Shanghai, China) and then iron fragmented at 95°C, followed by end repair and 5′ adaptor ligation. Reverse transcription was then performed with RT primers harboring 3′ adaptor sequences and randomized hexamers. After cDNA was purified and amplified, 200–500 bp PCR products were purified, quantified, and stored at −80°C until sequencing. cDNA clusters were generated and sequenced using an Illumina HiSeq2000 platform following the manufacturer’s instructions. Based on the SNPs and Indels information of the parents, we compared the genes with different expressions.

Based on the cDNA sequences for the candidate genes, Primer 5.0 software was used to design primers in a specific region at the 5′ end or 3′ end of a gene sequence for quantitative RT-PCR (qRT-PCR) analysis (Supplementary Table 2). For reverse transcription, 5 × All-In-One Mastermix (Abm, Canada) was used on root RNA. The resulting cDNA was used as a template for quantifying the expression of candidate genes by qRT-PCR on each biological sample with three technical replicates, and the internal reference gene used was *GbUBQ7*. Real-time PCR amplification was performed on an ABI 7500 Fast system. A BrightGreen 2 × qPCR Mastermix kit (Abm, Canada) was used according to the provider’s instructions. In a total amplification volume of 20 μL, the PCR reaction program was as follows: predenaturation at 94°C for 30 s, followed by 40 cycles of denaturation at 95°C for 5 s, annealing at 57°C for 5 s, and extension at 72°C for 34 s. The results were used for relative quantitative analysis via the 2^–ΔΔCt^ method (Tanino et al., 2017).

### Virus Induced Gene Silencing Analysis of *GbCML* in Cotton

Tobacco rattle virus (TRV)-based VIGS was performed in cotton as described previously (Liu and Page, 2008). The pTRV1, pTRV2, and pTRV2 derivatives harboring specific regions of *GbCML* were transformed into *Agrobacterium tumefaciens* strain GV3101 by electroporation. The primers used for *GbCML* fragment amplification are listed in Supplementary Table 2. Seven-day-old seedlings of the resistant parent 06-146 were transformed with a mixture of Agrobacterium cultures harboring pTRV1 with pTRV2, or its derivative plasmids. After the completion of inoculation, the 06-146 seedlings were washed with deionized water to remove excess agro-bacterial inoculum and grown at 25°C under a 16-h/8-h light/dark cycle in an environment-controlled growth chamber. After 2 weeks of cultivation, the plants were inoculated with FOV7 inoculum, as described earlier. The experiments were performed with at least 18 seedlings per treatment and were repeated three times. DSR was assessed for each seedling, as described earlier.

## Results

### Disease Severity Rate in Parents and Recombinant Inbred Lines

**FIGURE 1:**
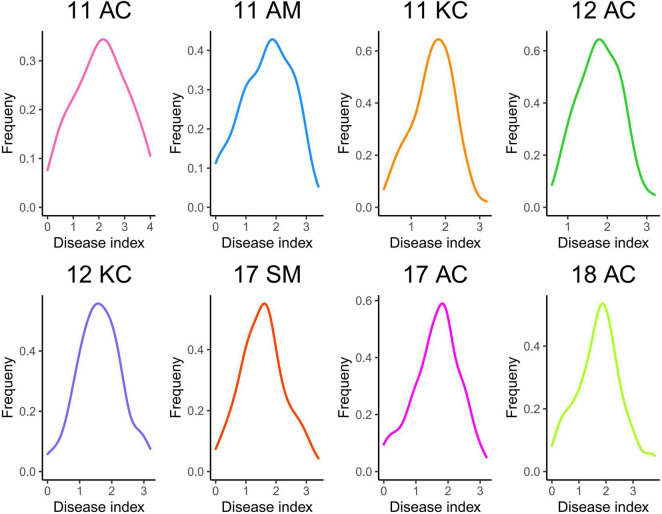
Frequency distribution of disease severity rating in 110 RILs tested in different environments.

Across the eight tests, the DSR ranged 0–0.8 for 06-146 and 1.2–3.0 for Xinhai 14, and Xinhai 14 had a significantly higher (*P* < 0.01) DSR than 06-146 in each test (Table 1). For the RIL population, the DSR ranged from 0 to 3.2–3.4 in six tests and as high as 3.8–4 in two tests. There were RILs that had significantly higher DSR and were therefore more susceptible to FOV7 than the susceptible parent, indicating transgressive segregation toward susceptibility in the RIL population. However, results in skewness and kurtosis in each test showed that the DSR followed a normal distribution in the RIL population (Figure 1 and Table 1). An analysis of variance (ANOVA) showed that the broad heritability estimates (i.e., the percentage of the total phenotypic variance accounted by the genotypic variance) of FOV7 resistance as measured by DSR in this RIL population ranged between 82.93 and 89.58%, indicating that the FOV7 resistance was highly heritable in this RIL population (Supplementary Table 3). The two-way analysis of variance (ANOVA) revealed highly effects of genotypes, environment and genotype × environment interaction on DSR. Most importantly, genotype explained the most variance while the genotype × environment interaction was accounted for more than 27% of the variation in each of the DSR (Supplementary Table 3).

**TABLE 1:** Disease severity rating in Xinhai 14, 06-146 and their RIL population of 110 lines.

Env	Parent			RILs					
	Xinhai 14	06-146	Diff	Mean	Min	Max	SD	Skewness	Kurtosis
11AC	2	0.6	**	2.08	0	4	2.09	–0.04	−0.87
11AM	1.8	0.4	**	1.69	0	3.4	1.69	–0.25	−0.79
11KC	1.6	0.2	*	1.64	0.2	3.2	1.64	–0.23	−0.37
12AC	3	0.8	***	1.81	0.6	3.2	1.81	0.08	−0.48
12KC	2	0.4	**	1.62	0	3.2	1.62	0.04	−0.22
17SM	1.2	0	**	1.56	0	3.4	1.56	0.19	−0.33
17AC	2	0.6	**	1.64	0	3.2	1.64	–0.3	−0.36
18AC	1.8	0.4	**	1.74	0	3.8	1.74	0.1	−0.17

11AC: Disease grade in the adult stage in Alar in 2011; 11KC: disease grade in the adult stage of Korla in 2011; 12AC: disease grade in the adult stage in Alar in 2012; 12KC: disease grade in Korla in 2012; 11AM: disease level in the seedling stage in Alar in 2011; 17SM: disease level in the indoor seedling stage in 2017. 17AC: disease grade in the adult stage in Alar in 2017. 18AC: disease level in adult plants in Aksu in 2018. Diff, difference. *P < 0.05; **P < 0.01; ***P < 0.001. SD, standard deviation.

The influence of genotype and environmental factors on plant traits has always been an obstacle in the process of breeding. Most traits are affected by both environmental and genetic factors. Understanding the mechanism of disease resistance is still a challenge in cotton breeding. Variance analysis of DSR in 8 environments showed that it was significantly affected by genotype, environment and genotype × environment interaction. Therefore, more attention should be given to changes in the planting environment to reduce the impact of environmental factors on cotton disease resistance in cotton production (Table 2). Because of the significant difference in DSR between the two parents and significant variation in DSR within the RIL population, further QTL mapping of FOV7 resistance is warranted. Because of the significant difference between the two parents and significant variation within the RIL population in DSR, further QTL mapping for FOV7 resistance was warranted.

**TABLE 2:** ANOVA for DSR traits across multiple environments.

Traits	Source	Df	Sum-Sq	Mean-Sq	*F*-value
DSR	Genotypes	109	127.6456	1.1711	5.903**
	Environment	7	40.0656	5.7237	160.548**
	genotype × environment	763	852.9738	1.1179	1.442**

** indicates significance at P < 0.01.

### Statistics of DNA Resequencing Data

The sequencing depth for Xinhai 14 and 06-146 was 13 × (30.95 Gbp) and 16 × (38.06 Gbp), respectively, with an average sequencing depth for the parents of 14.5 × and 97% of the genome coverage for the reference genome Hai 7124. The average sequencing depth for the RILs was 7 × (with an average of 17.65 Gbp per RIL) with average genome coverage of 95% (Supplementary Table 4). The Q30 value exceeded 90%, and the GC content was greater than 34.72% (Supplementary Table 5).

### Identification of Single-Nucleotide Polymorphisms Between the Two Parents and Construction of a High Density Linkage Genetic Bin Map

**FIGURE 2:**
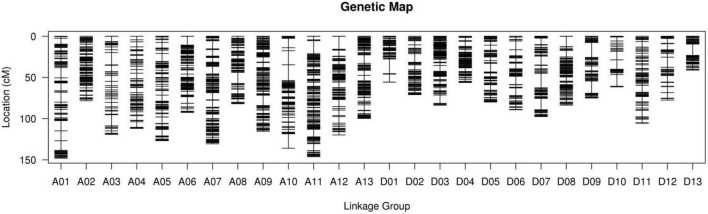
The distribution of SNP markers in the 26 linkage groups/chromosomes.

Using clean reads, a total of 933,845 SNPs were divided into 3,627 bins distributed on 26 chromosomes (Figure 2). The genetic map had a total genetic mapping distance of 2,483.17 cM (Table 3). The average number of bins in each chromosome was 138.5, with D13 having the fewest bins (53) and A06 the most bins (342). The average genetic distance for each chromosome was 95.51 cM, with D13 the shortest (41.17 cM) and A01 the longest (148.04 cM). The average genetic distance between two neighboring bins was 0.79 cM, and the largest gap between bins (19.3 cM) resided on A10 and D13. However, most of the gaps (96.71%) were less than 5 cM.

**TABLE 3:** Detailed information on the SNP markers in the genetic map.

Chr	No. SNP markers	No. bin markers	Total distance (cM)	Average distance (cM)	Max gap (cM)	Gaps < 5cM (%)
A01	18904	213	148.04	0.70	12.41	96.70
A02	25094	136	78.32	0.58	4.42	100.00
A03	873	75	119.06	1.61	14.97	90.54
A04	5385	81	110.86	1.40	6.67	93.75
A05	7788	141	126.76	0.91	9.80	95.71
A06	103552	342	92.44	0.27	10.76	99.41
A07	88947	198	130.55	0.66	9.80	96.45
A08	46482	178	81.89	0.46	13.38	98.87
A09	14426	195	115.37	0.59	12.61	98.45
A10	14795	105	135.91	1.31	19.30	95.19
A11	20501	236	146.22	0.62	15.90	98.72
A12	11927	145	119.78	0.83	15.83	95.83
A13	33777	192	99.73	0.52	10.24	97.91
D01	11723	105	55.68	0.54	17.76	98.08
D02	15850	110	70.88	0.65	10.48	98.17
D03	22293	163	83.75	0.52	10.08	98.77
D04	34639	153	56.17	0.37	6.09	99.34
D05	10251	89	79.87	0.91	10.48	96.59
D06	8743	88	89.21	1.03	10.57	94.25
D07	7078	96	97.56	1.03	12.62	95.79
D08	19467	138	83.58	0.61	18.12	98.54
D09	20960	110	74.99	0.69	14.17	97.25
D10	7865	70	61.44	0.89	8.50	95.65
D11	14638	131	105.48	0.81	12.22	96.92
D12	1806	58	77.46	1.36	13.72	89.47
D13	2920	53	41.17	0.79	19.30	98.08
Total	570684	3601	2483.17	0.79	19.30	96.71

### Collinearity Analysis Between the Linkage Map and the Physical Map

A collinearity analysis was performed between the positions of the same SNPs on the reference genome in physical distance and the linkage map in genetic distance (Supplementary Figure 1). An overall high collinearity was observed. Except for chromosomes A12 and D05 with a Spearman correlation coefficient below 0.9, the correlation coefficient was higher than 0.9 for each chromosome including 0.99 for D13, with an average of 0.96 (Supplementary Table 6). These results showed that the order of most of the SNP markers in the linkage map was highly consistent with their sequence positions on the reference Hai 7124 genome, indicating that the quality of the linkage map was highly reliable.

### Mapping of Quantitative Trait Locus for Resistance to FOV7

**FIGURE 3:**
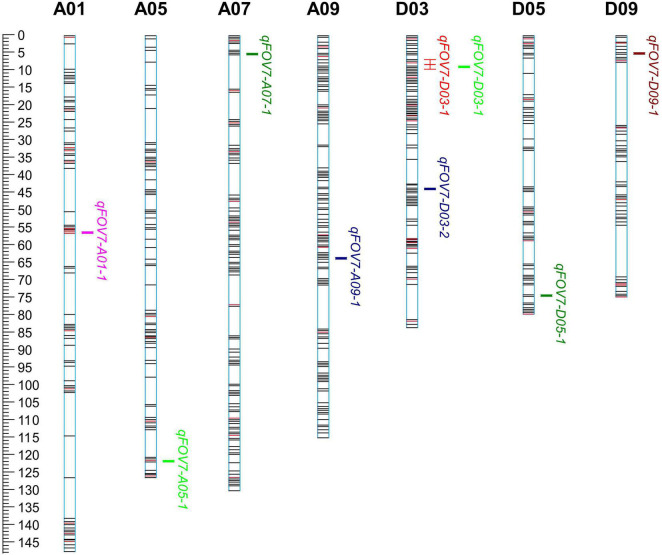
The chromosome distribution of QTLs for FOV7 resistance in a RIL population of *Gossypium barbadense*.

Nine QTLs for FOV7 resistance were detected on 7 chromosomes (A01, A05, A07, A09, D03, D05, and D09) (Table 4 and Figure 3), each of which (except for one) explained 3.5–19.7% of the phenotypic variation. Among the QTLs, 7 could be detected in only one environment, showing obvious environmental specificity and that the mode of action and expression of these QTLs changed greatly. QTLs detected in a single environment might be due to a low rate of phenotypic contribution, genetic effects, or environmental effects. Moreover, an unstable QTL can be considered promising for application if it has a strong effect. Nevertheless, such QTLs generally are not used.

**TABLE 4:** Summary of FOV7 resistance QTLs identified in different environments.

QTL name	Environment	Left marker	Right marker	Peak marker	LOD	ADD	PVE	Physical interval (bp)	Peak (bp)
*qFOV7-A01-1*	SM17	Block89	Block91	Block91	3	0.225	8.25	24580145–30697039	29066170–30697039
*qFOV7-A05-1*	AC17	Block1451	Block1453	Block1452	2	0.077	1.04	98049550–98784557	98078364–98663351
*qFOV7-A07-1*	AM11	Block2022	Block2027	Block2023	2	−0.165	4.03	1578481–2229225	1670902–1722583
*qFOV7-A09-1*	KC12	Block2986	Block2987	Block2987	2.5	0.162	10.14	62986283–63102116	63029492–63102116
*qFOV7-D03-1*	AC11	Block6644	Block6655	Block6648	4.8	−0.517	19.65	990342–3066473	1192410–1365428
*qFOV7-D03-1*	AC17	Block6649	Block6654	Block6654	2	−0.223	8.78	1367607–2774877	1891587–2774877
*qFOV7-D03-2*	KC12	Block6766	Block6767	Block6766	2.5	−0.113	4.93	21521563–29613781	21521563–22003728
*qFOV7-D05-1*	AM11	Block7246	Block7250	Block7246	2	0.154	3.49	27910706–29982056	27910706–28037736
*qFOV7-D09-1*	AC18	Block8062	Block8065	Block8063	2	−0.328	11.40	1981138–2462936	2312126–2421587

*Add and PVE represent the additive effect and phenotypic variation explained by the QTL, respectively.*

The widest range of phenotypic variation was observed in qFOV7-D03-1. A common QTL, qFOV7-D03-1, was identified on D03 in AC11 and AC17. A genome-wide association study (GWAS) showed that qFOV7-D03-1 was located on chromosome D03 chromosome and had a length of 1.97–2.37 Mb in *G. hirsutum*, consistent to the finding of Liu. This result strongly suggested that qFOV7-D03-1 is the main QTL for resistance to Fusarium wilt in G. barbadense. Therefore, qFOV7-D03-1 was considered to be a stably inherited QTL for follow-up studies related to FOV7 resistance.

### Identification of Candidate Genes for *qFOV7-D03-1*

**FIGURE 4:**
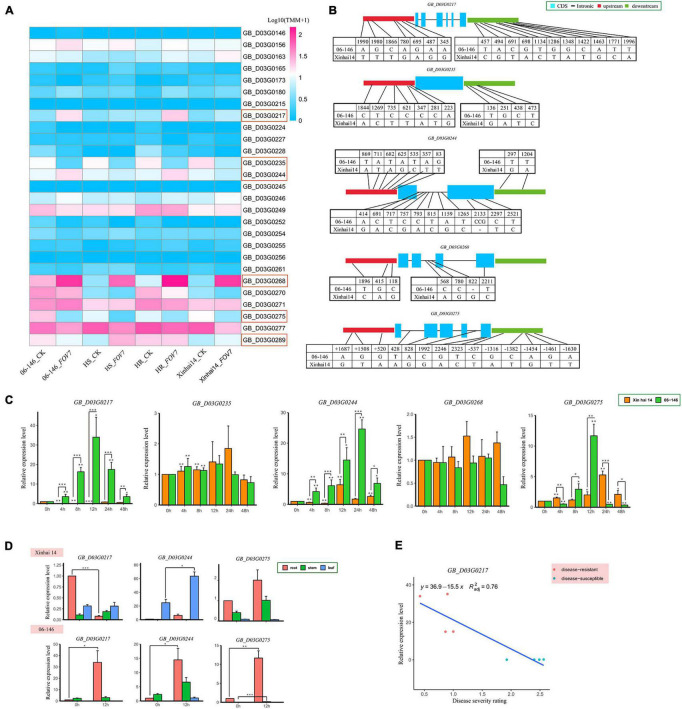
**(A)** Expression analysis of 27 genes from the parent and RIL populations under FOV7 stress based on transcriptome data. **(B)** SNP and Indel information between Xinhai 14 and 06-146. **(C)** Expression profiling of five candidate genes related to FOV7 resistance. The error bars represent the means of three replicates ± SEs. Statistically significant differences from the control group are indicated with *P < 0.05; **P < 0.01; and ***P < 0.001. **(D)** Tissue-specific expression analysis of three candidate genes related to FOV7 resistance. The error bars represent the means of three replicates ± SEs. Statistically significant differences from the control group are indicated as *P < 0.05; **P < 0.01;***P < 0.001. **(E)** Expression patterns of the *GB_D03G0217* gene in apical organs of eight lines inoculated with FOV7, detected by qRT–PCR; red and blue dots indicate four FOV7-resistant lines and four FOV7 susceptible lines, respectively.

Within the *qFOV7-D03-1* interval between 0.99 and 3.06 Mb on chromosome D03, 161 putative genes were predicted. Of these genes, 105 had annotation information (Supplementary Table 7). Through examining the transcriptome data of the parents and selected highly resistant (HR) and highly susceptible (HS) RILs within the RIL population, only six genes (*GB_D03G0217, GB_D03G0235, GB_D03G0244, GB_D03G0268, GB_D03G0275*, and *GB_D03G0289*) showed significant differential expression between the parents and between the HR and HS groups of RILs (Figure 4A and Supplementary Table 8). A comparison of the sequences between the two parents showed that upstream, downstream, exon, or intron regions in five genes (*GB_D03G0217, GB_D03G0235, GB_D03G0244, GB_D03G0268*, and *GB_D03G0275*) contained SNPs/Indels (Figure 4B).

To further study possible roles of these five candidate genes (with differences in expression levels and DNA sequences between the two parents) in FOV7 resistance, qRT-PCR was used to compare the transcription levels in the roots of the two parents at the seedling stage during different time points after FOV7 inoculation. Genes except for *GB_D03G0235* and *GB_D03G0268* exhibited increased expression in the roots after inoculation, as compared to the non-inoculated control (Figure 4C). However, only three genes (*GB_D03G0217*, *GB_D03G0244*, and *GB_D03G0275*) showed significant differential expression during the same period following inoculation between the two parents. The results suggest possible roles of these three genes in the response to FOV7 infection in *G. barbadense*.

To understand if these three genes are root-specific, their expression levels were compared among roots, stems and leaves. Results showed that their expression was generally the highest in the roots, both before and after FOV7 inoculation (Figure 4D). Interestingly, these three genes exhibited significant expression changes in different tissues before and after FOV7 inoculation. Among these three genes, only *GB_D03G0217* showed significant changes in the roots but with opposite expression patterns between the two parents and was therefore chosen as the possible candidate gene for the QTL.

To further study the involvement of the *GB_D03G0217* after *G. barbadense* was subjected to FOV7 infection, four FOV7-resistant lines and four susceptible lines were compared (Figure 4E). Results showed that the expression of *GB_D03G0217* was significantly higher in the resistant lines than in the susceptible lines. The linear regression between the DSR and the expression level in the eight lines was significant (*r* = 0.872, *P* < 0.01).

### Virus Induced Gene Silencing Analysis of *GbCML* in Cotton

**FIGURE 5:**
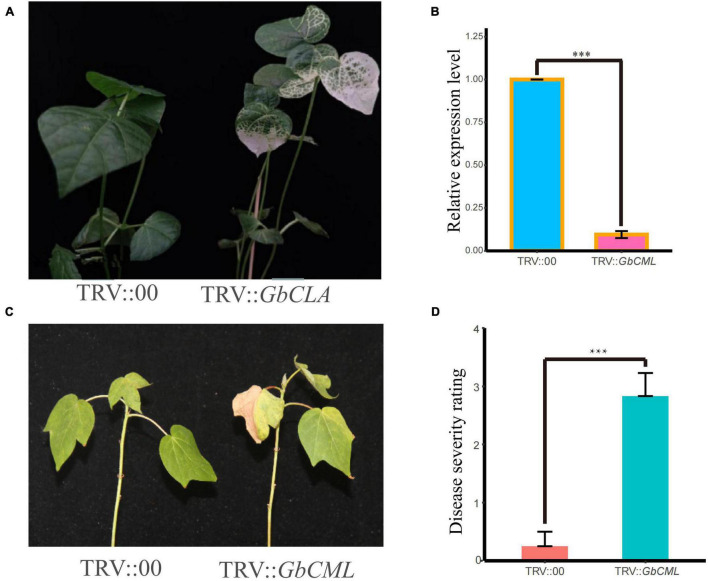
Effects of silencing of *GbCML* on 06-146 susceptibility to FOV7. Two weeks after infiltration, seedlings were inoculated with FOV7. **(A)** Seven day-old cotton plants were infiltrated with Agrobacterium carrying TRV:*GbCLA*. The photographs were taken at 2 weeks after infiltration. **(B)** qRT-PCR for detection of silencing efficiency. **(C)** Representative seedlings of control (CK) and silenced plants after inoculation with FOV7 at 20 days post-inoculation (dpi). **(D)** Responses of control (CK) (TRV:00) and silenced (TRV:*GbCML*) plants to the FOV7 at 20 dpi. Disease severity rating (DSR) in CK and silenced plants each with eighteen experimental replicates. The DSR were measured at 20 dpi. Error bars represent the standard deviation of eighteen biological replicates; asterisks indicate statistically significant differences, as determined by *t*-test (*P* < 0.001).

To further verity the function of *GB_D03G0217* in relation to FOV7 resistance, a bioinformatics analysis indicated that it encodes a calmodulin-like (CML) protein, named *GbCML* here. As shown in Figure 5A, VIGS plants infiltrated with Agrobacteria carrying *GbTRV1* + *GbCML* exhibited an albino phenotype on newly developed true leaves after infiltration, indicating that the VIGS system worked efficiently under our experimental conditions, as expected. The qRT-PCR analysis showed that the expression of *GbCML* in VIGS plants was significantly lower than that in control plants with the empty vector (Figure 5B). To further understand the relationship between changes in *GbCML* expression and FOV7 resistance in VIGS plants, 18 VIGS plants were evaluated for their resistance to FOV7 (Figures 5C,D). The VIGS plants exhibited FOV7-associated foliar symptoms including necrosis and yellowing (Figure 5C), with a significant higher DSR (Figure 5D). Therefore, suppression of the *GbCML* gene expression in the VIGS plants of the resistant parent 06-146 led to increased susceptibility to FOV7. The results suggested that *GbCML* played a role in defending cotton against FOV7.

## Discussion

In this study, we resequenced 110 *G. barbadense* RILs and their two parents and constructed a high-density genetic map in a *G. barbadense* RIL population for the first time based on 933,845 high-quality SNP markers. The linkage map had a total genetic distance of 2,483.17 cM for 26 linkage groups (i.e., chromosome pairs). The genetic variation within *G. barbadense* is known to be low (Zhang et al., 2014). Because of the close genetic relatedness of the two parental lines, which were developed in Xinjiang, China, there were chromosomal regions with minimal sequence variation, leading to large gaps between bins. However, as expected, the RIL population showed a higher recombination rate and higher position resolution than the F_2_ population due to many generations of self-pollination to break tight linkages. Although the RIL population was not large, the DSR trait was normally distributed in each of the eight tests, indicating that the RIL population was suitable for identifying QTLs for FOV7 resistance in *G. barbadense*. As a result, nine QTLs for FOV7 resistance, including one common QTL (*qFOV7-D03-1*) on chromosome D03, were identified. This study represents the first investigation of FOV7 resistance using a RIL population evaluated in multiple tests for QTL mapping in *G. barbadense*.

Wang et al. (2009) was the first to use molecular mapping technology to identify four QTLs, including one major gene (FwR) on D03, for FOV7 resistance in early segregating populations (F_2:3_) of *G. hirsutum* based on SSR markers. Most recently, Liu et al. (2021) reported that *Gh_D03G0209* (named *GhGLR4.8*) on D03 encoding a GLUTAMATE RECEPTOR-LIKE (GLR) protein was the candidate gene for the resistance gene Fov7 within the 1.97–2.37 Mb region based on a GWAS of 290 Chinese *G. hirsutum* accessions and CRISPR/Cas9-mediated knockout analysis. The region containing FOV7 is within the region (0.99–3.01 Mb) of the common QTL *qFOV7-D03-1* identified in this study. Although *GhGLR4.8* has the highest homology (99.84%) to *GB_D03G1991* on the same chromosome, *GB_D03G1991* is not within the QTL region for *qFOV7-D03-1*. Therefore, its role in conferring resistance to FOV7 in this *G. barbadense* RIL population was ruled out. The genetic bases of resistance between *G. barbadense* and *G. hirsutum* are likely different.

In this study, a detailed comparative analysis of gene expression and DNA sequences within the *qFOV7-D03-1* region between the two parents revealed only five candidate genes. Through further qRT-PCR of different tissues between the two parents and roots after inoculation of FOV7 between two groups (resistant vs. susceptible) of lines, *GB_D03G0217* was identified as the candidate gene for verification using VIGS. *GB_D03G0217* was homologous to the gene *AT3G50360* encoding a calmodulin (CaM)-like (CML) protein in Arabidopsis. It is currently known that CML and CaM proteins are primary Ca2 + sensors regulating cellular functions in response to environmental cues, including gene expression during plant immune responses (Chiasson et al., 2005; Cheval et al., 2013; Zhu et al., 2017; Bredow and Monaghan, 2019).

To date, many studies have focused on yield and fiber quality traits in *G. barbadense*, while few studies have focused on resistance to FOV7. Due to the planting pattern of cotton in the field, disease resistance has become the main problem and challenge in cotton breeding. In our study, stable QTLs and candidate genes related to FOV7 resistance were obtained by QTL mapping in *G. barbadense*. Although 7 QTLs could be detected in only one environment, they showed obvious environmental specificity. Unexpected results might be obtained if we further apply multiomics. Although only a small number of stable QTLs were detected, unstable QTLs can also be used to select some disease-resistant varieties for specific areas. To further improve the location resolution for in-depth analysis, a larger genetic population needs to be developed with the stable QTL region. This study laid a foundation for improving disease resistance, breeding efficiency and the development of molecular markers in cotton breeding.

In summary, nine QTLs related to FOV7 resistance were identified, including *qFOV7-D03-1* on chromosome D03, in two experiments. However, QTL stability was influenced by the environment. Through a combined analysis of QTL linkage mapping and gene expression, the CML gene (*GB_D03G0217*) gene on D03 was identified as a candidate gene likely conferring resistance to FOV7 in *G. barbadense*. VIGS showed that resistance to FOV7 in *G. barbadense* was positively regulated by *GBCML*.

## Data Availability Statement

The data presented in the study are deposited in the NCBI repository, accession number PRJNA798892.

## Author Contributions

WH, QJC, and YQ conceived and designed the experiments. WH, JYZ, DL, YW, QC, QZ, XL, and XD collected public datasets and performed the experiments. WH, JYZ, JFZ, XD, AG, QJC, and YQ analyzed the data. WH, JYZ, and JFZ wrote the manuscript. WH, JFZ, QJC, and YQ revised the manuscript. All authors read and approved the final manuscript.

## Conflict of Interest

The authors declare that the research was conducted in the absence of any commercial or financial relationships that could be construed as a potential conflict of interest.

## Publisher’s Note

All claims expressed in this article are solely those of the authors and do not necessarily represent those of their affiliated organizations, or those of the publisher, the editors and the reviewers. Any product that may be evaluated in this article, or claim that may be made by its manufacturer, is not guaranteed or endorsed by the publisher.

## Footnotes

1
https://www.cotlook.com/wp-content/uploads/2020/01/Long-Staple-Annual-review-2019.pdf

## Supplementary Material

Supplementary Figure 1 Genetic linkage map and genome collinearity map.
