# Supplementary figures and images for: Quantitative Trait Locus Mapping and Identification of Candidate Genes for Resistance to Fusarium Wilt Race 7 Using a Resequencing-Based High Density Genetic Bin Map in a Recombinant Inbred Line Population of Gossypium barbadense
Source: Front Plant Sci. 2022 Mar 10;13:815643. doi: 10.3389/fpls.2022.815643 (PMC8965654; doi:10.3389/fpls.2022.815643)

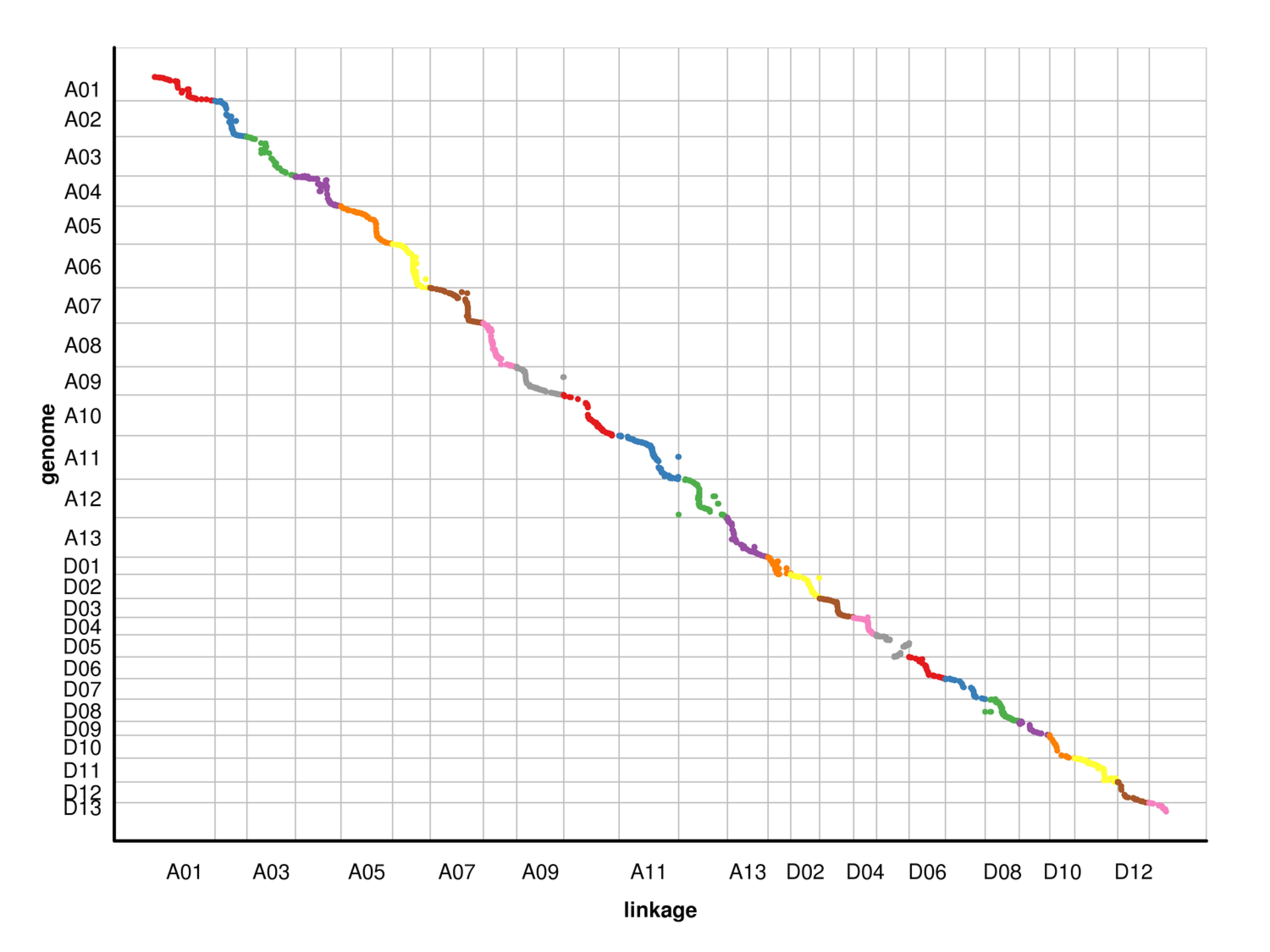

Supplement: Supplementary Figure 1 — Genetic linkage map and genome collinearity map. [file Data_Sheet_1.ZIP › Supplementary information/Fig S1.jpg]
